# Ecological risk assessment of landscape in arid area watersheds under ecological water conveyance: A case study of Taitema Lake

**DOI:** 10.1016/j.heliyon.2024.e29575

**Published:** 2024-04-14

**Authors:** Zhentao Lv, Shengyu Li, Xinwen Xu, Jiaqiang Lei, Zhongmin Peng

**Affiliations:** a State Key Laboratory of Desert and Oasis Ecology, Xinjiang Institute of Ecology and Geography, Chinese Academy of Sciences, Urumqi, 830011, Xinjiang, China; b University of Chinese Academy of Sciences, Beijing, 100049, China; c StarWiz Technology Co., Ltd, Beijing, 100044, China

**Keywords:** Terminal lake, Ecological restoration, Ecological risk index, Ecological water conveyance

## Abstract

The Taitema Lake Basin serves as an ecological barrier in the south of the Tarim Basin, connecting with the Qiemo, Ruoqiang, and Milan oases, collectively preventing the expansion and merging of the desert, specifically inhibiting the convergence of the Taklamakan Desert and Lop Nur. In recent years, with changes in the natural environment and an increase in water usage, the downstream flow of the Tarim River has decreased, leading to the gradual drying up of Taitema Lake and exacerbating desertification, resulting in frequent sandstorms. Subsequently, under the influence of ecological water transfer projects, Taitema Lake has gradually recovered, and the ecological environment has improved. This study focuses on Taitema Lake and its nearby regions, constructing the Regional Landscape Ecological Risk Index (ERI) to assess landscape ecological risks before and after ecological engineering and determine ecological benefits. The results indicate that the EWC (Ecological Water Conveyance) project effectively supplements water in the intermediate and lower courses of the Tarim River and the terminal lakes, significantly bolstering ecological conditions in the lake basin and reducing risks. However, the current EWC project is relatively extensive, and the water reaching the Tarim River and Taitema Lake depends entirely on the surplus water from upstream production and daily life. Additionally, the distribution of downstream water depends entirely on natural topography, leading to uneven spatiotemporal distribution of water resources and significant evaporation losses. Rational hydraulic engineering measures should be taken to re-plan the distribution of rivers and lakes, achieving the maximum ecological benefits of the EWC project.

## 1 Introduction

In arid regions, oases are generally distributed around rivers and lakes. Terminal lakes are not only the main water source in arid areas but also serve as ecological barriers for oases. They have a series of functions such as improving local climate, reducing dust storms, blocking desert expansion, and protecting biodiversity[1]. However, due to the unreasonable use of water resources and the fragility of the regional ecological environment, oases and rivers/lakes in arid areas are very fragile. Events such as oasis shrinkage, river drying up, and lake drying are becoming more common. This leads to reduced vegetation, frequent dust storms, and desert expansion. This seriously affects the ecological stability of oases in arid areas, and the basin environment faces severe ecological security issues, posing a serious threat to human health and survival[2]. In response to this situation, the Chinese government launched a series of ecological water transfer projects in arid regions such as the Tarim River, Heihe River, and Shiyang River in 2000. By transferring water across basins or replenishing tributaries of dried-up or interrupted rivers, the aim is to restore the dried-up vegetation, degraded ecosystems, and shrinking oases around the dried-up river channels[[3], [4], [5]]. This demonstrates that ecological water transfer has significant effects on the restoration of inland river ecosystems, and vegetation restoration in many basins has been widely evaluated[6,7]. Other countries have also implemented large-scale water diversion projects[8,9]. Ecological water transfer projects generally use methods such as flood irrigation, which significantly replenish groundwater and reduce soil salinization. Water transfer is mainly carried out through ecological gates, agricultural gates, and river channels to introduce water into degraded areas, aiming to restore and rebuild ecosystems[10,11]. Ecological water transfer increases the depth of groundwater in the basin, improves water quality in terminal lakes and downstream rivers, and significantly restores surrounding vegetation.

The Tarim River Basin is the largest inland river basin in China, nourishing numerous oases and vast forests of Populus euphratica along the eastern edge of the Tarim Basin for a long time, forming a “green corridor” that prevents the expansion of the Taklimakan Desert[12]. However, due to the fragile local ecological environment[13] and the unreasonable utilization of water resources in the basin since the 1960s, downstream water flow has significantly decreased[14]. After the construction of the Daxihaizi Reservoir in 1972, the Tarim River dried up 321 km downstream from the reservoir, the terminal lake Taitema Lake dried up, riverbank vegetation died in large numbers, desert expansion occurred, and the ecological environment deteriorated[15]. Since 2000, ecological water conveyance (EWC) has been implemented in the Tarim River Basin, with water being diverted 21 times over 20 years to supplement downstream water flow[16]. Today, the effects of EWC are significant: vegetation in the middle and lower reaches of the Tarim River has regrown, Taitema Lake has restored its water source, and the basin's ecological environment has improved. Although the landscape of the basin and many studies have proven the effectiveness of EWC, there are still some challenges in its allocation and implementation. In arid regions, especially in the Taklimakan Desert area, potential evaporation far exceeds precipitation. Maintaining large areas of surface water increases water loss, and a large amount of ecological water is lost through evaporation without being used to replenish groundwater or support vegetation growth. Furthermore, data from the drainage of the Daxihaizi Reservoir shows that the water volume for EWC is entirely dependent on upstream water inflow, without considering the actual ecological water demand and balance of ecological water in the basin[17]. EWC projects aim to restore water bodies and vegetation landscapes, maintain landscape stability, reduce landscape ecological risks, and improve the ecological environment by supplementing water to the middle and lower reaches of the basin. Therefore, accurately assessing the landscape ecological risks in EWC implementation areas is an important indicator of evaluating the effectiveness of EWC.

Nowadays, landscape ecological risks are increasingly being used in ecological restoration and environmental management[18]. Ecological risks reflect the intensity of external risks faced by ecosystems and their ability to resist these risks[19]. These factors may include natural disasters, climate change, pollution, biological invasions, overexploitation, and resource overuse. The health and stability of ecosystems are crucial for the survival and development of human society. An increase in ecological risks may lead to severe environmental problems and have adverse effects on human health, economy, and society. From a landscape perspective, focusing on ecological risks can extend from single factors or single ecological environments to broader landscape scales[20,21]. Landscapes are not just simple assemblages of natural elements and human-made structures but complex ecological networks formed by the interaction and combination of these elements. Landscape ecology emphasizes the interactions between spatial patterns, ecological processes, and scales. Therefore, by assessing ecological risks from a landscape perspective, we can better understand the integrity and complexity of ecosystems, thus formulating more effective protection and management strategies. In previous studies on the effectiveness of EWC, some scholars have studied the effects of EWC implementation on changes in groundwater depth[[22], [23], [24]], hydrological and water quality changes[25], vegetation responses[26,27], vegetation physiological changes[28,29], and socio-economic changes[30]. However, most studies only focus on the response of single factors to EWC, lacking assessments and studies on the overall changes in the ecological environment of EWC regions. Different factors may have differences in response ranges and response mechanisms to EWC. By evaluating the spatiotemporal changes in landscape ecological risks before and after EWC implementation, we can more intuitively and comprehensively describe the scope, degree, and mechanism of EWC's impact on basin ecological environments. These results will provide scientific basis for adjusting the frequency of ecological water transfer, allocating water resources, and planning water transfer modes.

This study, conducted from the perspective of landscape ecology, aimed to analyze the spatiotemporal changes in landscape ecological risk induced by the Ecological Water Conveyance (EWC) in the Taitema Lake Basin by calculating the landscape ecological risk index before and after the implementation of EWC. The objectives of this study were as follows: (1) to analyze the land cover changes in the Taitema Lake Basin from 1986 to 2020, (2) to construct a landscape ecological risk assessment model suitable for the study area and calculate the landscape ecological risk index before and after the implementation of EWC in the study area, (3) to assess the spatial clustering of different levels of Ecological Risk Index (ERI) before and after EWC, as well as the landscape ecological risk index of different land cover types, and analyze the reasons behind them, (4) to evaluate the influence range and mechanism of surface water bodies on ERI. The results of this study can provide data and technical support for the sustainability, water conveyance modes, and optimization of landscape patterns of ecological water conveyance projects.

## 2 Materials and methods

### 2.1 Study area

**Fig. 1:**
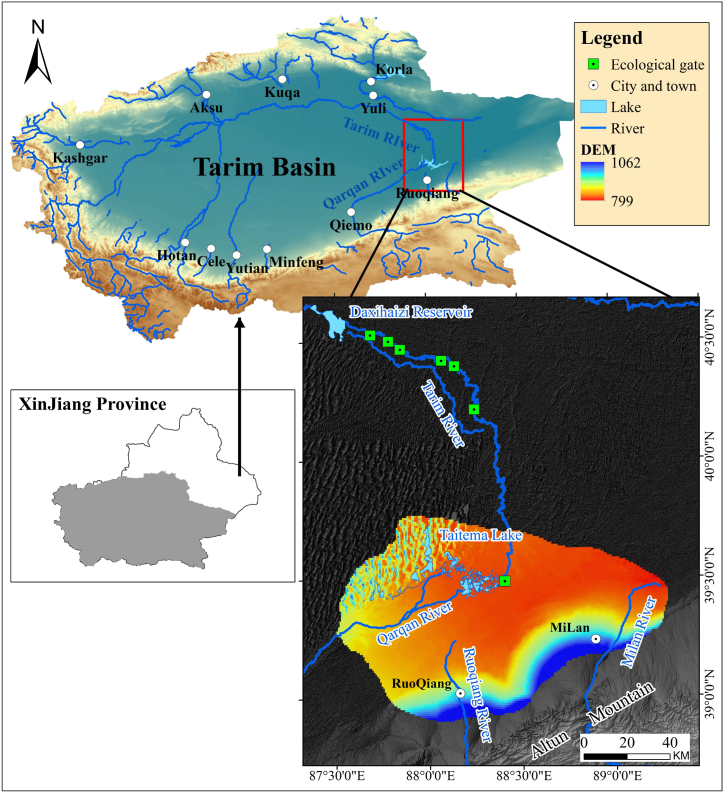
Location of the study area.

Taitema Lake, situated in the southeastern part of the Tarim Basin, is an endorheic lake at the tail end of the Tarim River and Qarqan River, bordered by the Taklamakan Desert to the west, the Kuruk Desert and Lop Nur to the east (Fig. 1). The Tarim Basin is one of the largest inland river basins in the world, and the Tarim River is China's longest inland river, originating from glacier meltwater and stretching 1321 km before ultimately flowing into Taitema Lake. Taitema Lake experiences an extremely arid continental climate with annual average precipitation of 55 mm, annual average evaporation of 2,870 mm, annual average temperature of 7.6 °C, and a total solar radiation of 119.2 kcal/cm³ [31,32]. Over the past four decades since the 1970s, due to natural and human factors, Taitema Lake has gradually dried up[33]. The disappearance of the lake and the decline in groundwater levels have caused a widespread reduction in wetlands and natural vegetation, contributing to the rapid proliferation of land desertification in the region. It wasn't until the initiation of an EWC project in 2000 that groundwater levels gradually recovered ([16]; Zhu et al., 2010). After two decades of concerted recovery efforts, the lake's water body has reemerged, again playing a pivotal role in supporting vegetation growth in the area.

### 2.2 Data sources

This study primarily relies on Landsat TM/OLI remote sensing images from 1986, 1991, 1995, 2001, 2005, 2009, 2016, and 2020 (http://earthexplorer.usgu.gov). We also referenced the 30 m annual land cover database for China from 1990 to 2021 (https://zenodo.org/records/5816591). Using the Random Forest classification method, we conducted image classification to generate a 30m-resolution land use type map for the study area.

### 2.3 Methods

#### 2.3.1 Landscape risk assessment model

Landscape ecological risk reflects the sensitivity of landscape patterns to external disturbances and the attributes that result in changes in landscape system structure, function, and characteristics due to a lack of adaptability. This study conducted an ecological risk assessment of the study area by calculating the Landscape Ecological Risk Index based on Landscape Disturbance Index (E_i_) and Landscape Vulnerability Index (V_i_). To enhance the accuracy of ERI and visualize ERI for different locations, we divided the study area into grids and calculated the ERI within each grid. This represents the ERI at the center of each grid. Subsequently, we utilized Kriging spatial interpolation technique to obtain the spatial distribution of ERI across the entire region. Building upon previous experience[34], the study area was divided into grids of 2.5 km by 2.5 km, resulting in a total of 1005 grid cells. The specific calculation process of ERI is as follows.

Landscape Disturbance Index (Ei) represents the degree of disturbance in different landscapes, primarily determined by human development activities[35]. E_i_ is obtained by superimposing the Landscape Fragmentation Index (C_i_), Landscape Isolation Index (N_i_), and Landscape Dominance Index (D_i_). The Landscape Fragmentation Index (C_i_) represents the degree of landscape fragmentation, the complexity of landscape structure, and the intensity of human disturbance, which is inversely proportional to the stability of the landscape. The Landscape Isolation Index (N_i_) refers to the degree of separation of each patch, with higher values indicating poorer overall connectivity and more scattered landscape distribution. The Landscape Dominance Index (D_i_) indicates the position of patches in the landscape, representing the extent to which patches influence the formation and change of landscape patterns.

(1) $$E_{i}={aC}_{i}+{bN}_{i}+{cD}_{i}$$

(2) $$C_{i}=\frac{n_{i}}{A_{i}}$$

(3) $$N_{i}=\frac{A}{{2A}_{i}}\sqrt{\frac{n_{i}}{A}}$$

(4) $$D_{i}=\frac{P_{i}+M_{i}}{4}+\frac{L_{i}}{2}$$

where n_i_ represents the number of patches in landscape i; A_i_ is the landscape area; A is the total area; P_i_ is the perimeter of landscape i; a, b, and c respectively represent the weights of C_i_, N_i_, and D_i_; a + b + c = 1. The weight values reflect the intensity of the ecological impact of different landscape disturbances. Among them, C_i_ is considered the most important, followed by N_i_ and D_i_. Based on previous research and our analysis[35,36], we assigned values of 0.5, 0.3, and 0.2 to a, b, and c, respectively.

The Landscape Vulnerability Index (V_i_) represents the vulnerability of the internal structure of an ecosystem[37], reflecting the resistance level of different landscape types to external disturbances. Based on previous research (X. [35,38]) and the specific conditions of the study area, the vulnerability of five landscape types has been determined, ranked from low to high vulnerability as follows: cropland = 5, impermeable surfaces = 4, grassland = 3, water bodies = 2, and wasteland = 1. Subsequently, the V_i_ of land use types is normalized according to the order of vulnerability.

The Landscape Loss Index (R_i_) represents the degree of natural attribute loss of different landscape types when subjected to natural and human disturbances. The formula is as follows:

(5) $$R_{i}=E_{i}\times V_{i}$$

where R_i_ represents the Ecological Loss Index of landscape i; E_i_ represents the Landscape Disturbance Index of landscape i; and V_i_ represents the landscape Vulnerability Index of landscape i.

(6) $$ERI=\sum_{i=1}^{m}\frac{A_{ni}}{A_{n}}R_{i}$$

where A_ni_ represents the area of type i within the n-sample area; A_n_ represents the total area of the n samples; and m represents the number of landscape types.

#### 2.3.2 Spatial autocorrelation analysis

Spatial autocorrelation analysis is a pivotal method in spatial statistics, providing a quantitative description of spatial correlation (Anselin, 1980; Moran, 1948). Global spatial autocorrelation employs the global Moran's I index to portray the distribution characteristics of an attribute across the whole region. The formula is as follows:

(7) $$global\;Moran^{\prime }s\;I=\frac{1}{\sum_{i=1}^{n}\sum_{j=1}^{n}W_{ij}}∙\frac{\sum_{i=1}^{n}\sum_{j=1}^{n}W_{ij}\left(x_{i}-\bar{x}\right)\left(x_{j}-\bar{x}\right)}{\sum_{i=1}^{n}\frac{{\left(x_{i}-\bar{x}\right)}^{2}}{n}}$$

where x_i_ and x_j_ are the variates x in adjacent paired spatial cell; x is the average values at n positions; W_ij_ is the element of binary spatial weight matrix W, and it can be constructed based on adjacency criteria or distance criteria to reflect the similarity of positions to spatial target.

In contrast, the local Moran's I index gauges the correlation degree of attribute values in neighboring spatial areas.

(8) $$local\;Moran^{\prime }s\;I=\frac{\left(x_{i}-\bar{x}\right)}{\frac{\sum_{i}{\left(x_{i}-\bar{x}\right)}^{2}}{n}}\sum_{j}W_{ij}\left(x_{j}-\bar{x}\right)$$

A positive global Moran's I index signifies the clustering of similar values within a region, while a negative index indicates the clustering of dissimilar values. Higher positive local Moran's I value denotes regions with similarly high or low values compared to their neighbors, whereas higher negative local Moran's I value highlights significant differences in location between a region and its surroundings.

#### 2.3.3 Geographically Weighted Regression

Geographically Weighted Regression (GWR) is a spatial statistical technique used to explore the relationships between variables in spatial data. Unlike traditional global regression models, GWR allows model parameters to vary spatially to better capture spatial non-stationarity and spatial heterogeneity present in geographic data. In GWR, regression coefficients are functions of geographic location, resulting in a specific set of coefficients for each observation point. This makes GWR particularly useful for studying spatial heterogeneity and local spatial relationships in geographic data. Using the GWR model to analyze the impact intensity of water bodies and human settlements at different geographical locations on ERI. The GWR formula is as follows.

(9) $$y_{k}=\beta_{0}\left(u_{k},v_{k}\right)+\sum_{i=1}^{n}\left(u_{k},v_{k}\right)x_{ki}+c_{k}$$

in the formulate, y_k_ is the value of ERI, x_ki_ is the distance to water/impermeable, n is the total number of spatial units involved in the analysis, c_k_ is the random error term, (u_k_,v_k_) is the spatial location of sample k, β_0_ (u_k_,v_k_) is the intercept at location k, and β_0_ (u_k_,v_k_)x_ki_ is the coefficient of the I the independent variable of sample k.

## 3 Results

### 3.1 Land use changes

**Fig. 2:**
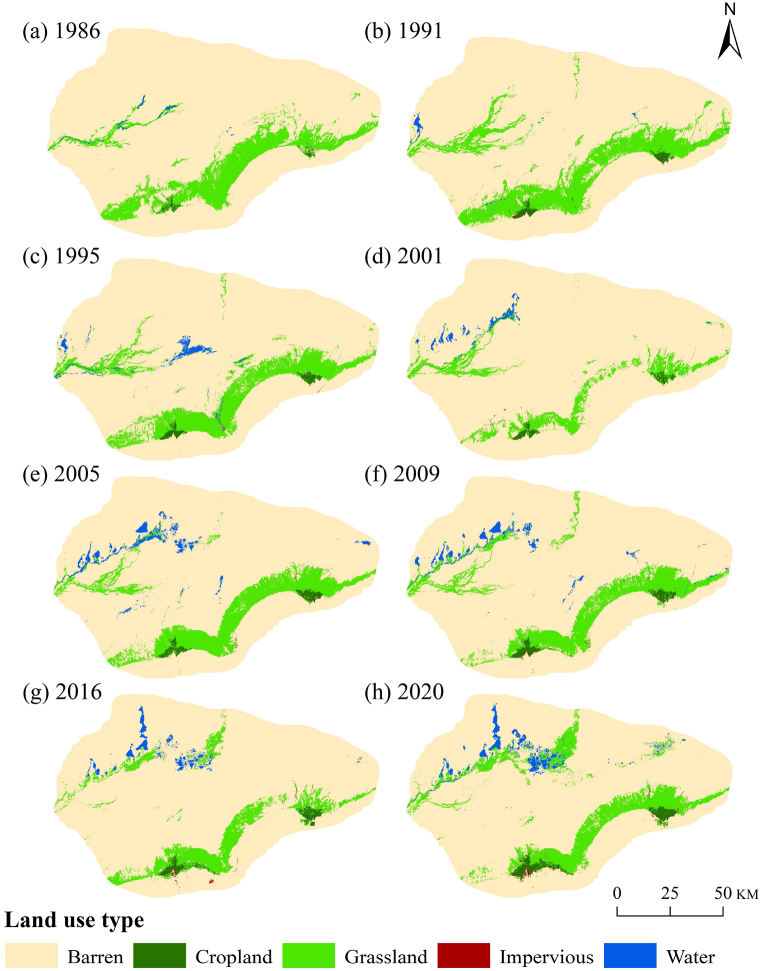
Change of landscape types in Taitema Lake Region during1986–2020.

**Fig. 3:**
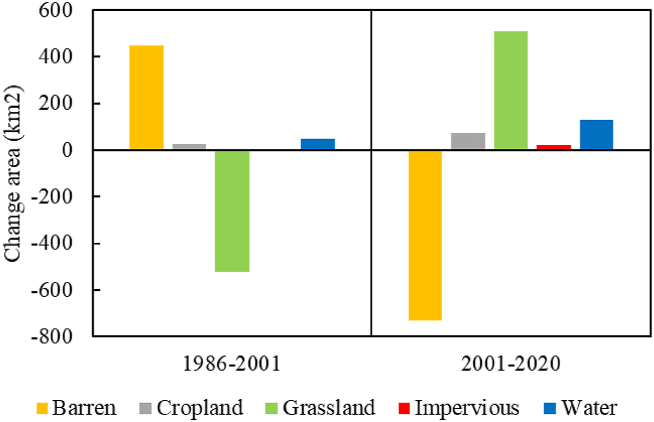
Area changes in LUCC in the Taitema Lake region for 1986–2001 and 2001–2020.

The LUCC of the study area underwent two stages of transformation (Fig. 2). The first stage, before 2000, saw the complete utilization or natural dissipation of the Tarim River water sources in the middle and upper reaches. During this period, no water reached Taitema Lake, and the lake's water source relied entirely on the Qarqan River from the west. The water area accounted for 0.24%–1.06 % of the study area. Most of the region was covered by deserts, exceeding 80 %. Vegetated areas were primarily grasslands (7%–12 %), followed by croplands (0.3%–0.68 %). Croplands were situated exclusively in the Milan Town and Ruoqiang County areas, while grasslands were mainly distributed around water bodies and on the alluvial plains of the Altun Mountains, forming a green barrier that obstructed the southeast expansion of the Taklimakan Desert. Simultaneously, from 1989 to 2002, the Qarqan River gradually changed its course northward, leading to a gradual reduction in grassland area between Milan Town and Ruoqiang County[39]. Consequently, during the period from 1986 to 2001, grassland in the study area decreased by 522 km^2^. In the second stage, which began after the initiation of the EWC in 2000, the study area experienced changes. From 2001 to 2020, the water body increased by 127 km^2^, accompanied by a reduction in the desert area by 731 km^2^, and an increase in grassland by 509 km^2^ (Fig. 3). Additionally, cropland and impervious surfaces increased by 72 km^2^ and 19 km^2^, with increments of 129 % and 365 %, respectively.

### 3.2 Characteristics of temporal and spatial variation in ERI

**Fig. 4:**
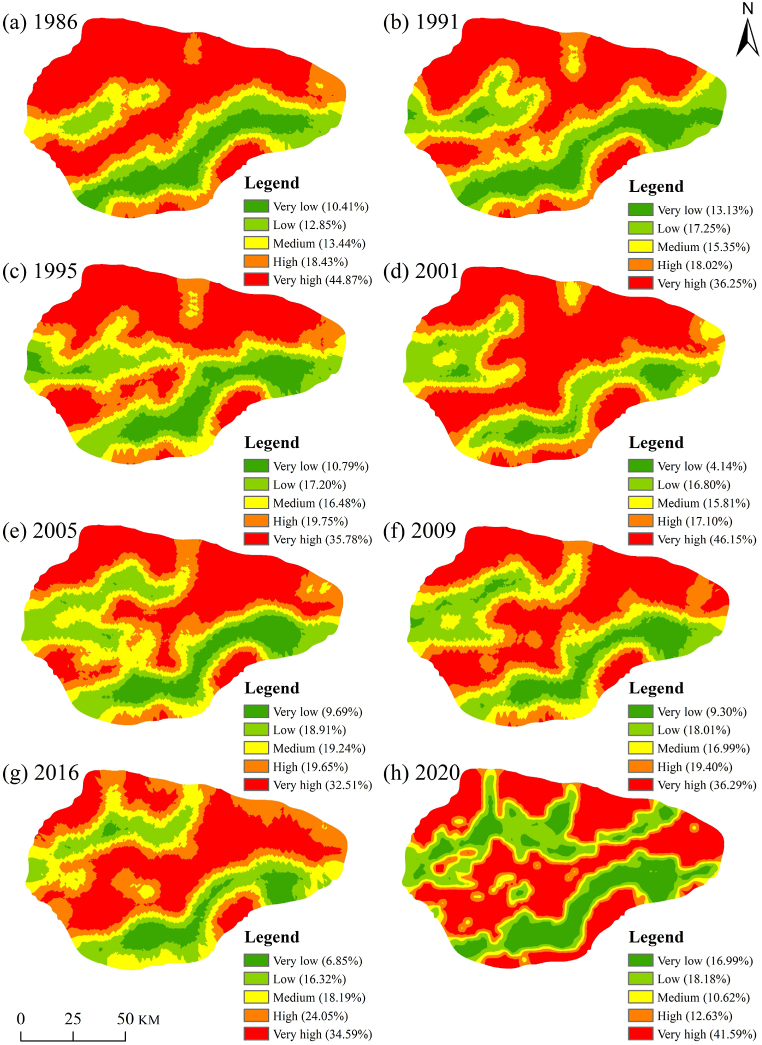
Map of the ERI in the Taitema Lake region for 1986–2020.

From 1986 to 2020, the mean ERIs for the study area were 3.74, 3.47, 3.53, 3.84, 3.46, 3.55, 3.6, and 3.44, respectively. This indicates that ERI in the study area increased before 2000 and a decreased trend from 2000 to 2020. Under the natural break method, the risk level was categorized as very low, low, moderate, high, and very high, as shown in Fig. 4.

From the results, it can be observed that ecological risk is very high in most parts of the study area. Single desert landscapes often correspond to higher ERI values. In contrast, landscape types with lower landscape vulnerability, such as grasslands, croplands, impervious surfaces, and proper landscape configurations, exhibit lower ecological risk. Fig. 4a shows that in 1986, the parts with very high ERI were mainly located in the northwestern Taklimakan Desert and the northeastern Lop Nur. The very low ERI parts were primarily situated in the oases of the Altun Mountains' foothills. This oasis comprises the eastern Milan Town, the central Ruoqiang County, and the western Qiemo County. The central part of the oasis had the lowest ecological risk. The low ERI parts were mainly distributed at the periphery of the oasis and downstream of the Qarqan River. Moderate ecological risk areas were predominantly found in the transitional zones between grasslands and deserts. The original Taitema Lake region, due to a continuous reduction in the flow of the Tarim River, leading to the river retreating northward, had very little vegetation growth and fell into the category of medium to high ecological risk. Starting from 1989, the Qarqan River began to change its course northward. From 1991 onward, it can be observed in Fig. 4b that parts of low and very low ERI downstream of the Qarqan River gradually expanded to the northwest. Due to the gradual northward shift of the Qarqan River away from the foothills of the Altun Mountains, the original oasis, with a diminishing water source, experienced an increasing ecological risk. By 2001, as the river's course alteration came to an end, the previously low ecological risk areas downstream of the Qarqan River had transformed into areas of medium to high and very high ecological risk. The new downstream riverbed and the emerging Kanglayka Lakes gradually transitioned to the parts of low and very low ERI. The ERI of the oases on the Altun Mountains' foothills also rose to the highest level. At this point, the very high ERI areas had increased from 44.87 % in 1986 to 46.15 %, while the very low ERI areas decreased from 10.41 % to 4.14 %.

In the 2001 autumn (from October 8th to November 9th), the Tarim River water was directed through a 14 km artificial channel below the Kurghan River to reach the Taitema Lake area. In the spring of 2003 (before April 1st), the Qarqan River water passed under National Highway 218. During the summer of 2003 (from May 7th to May 23rd), the Qarqan River water merged with the Tarim River water. After the end of 2010, due to increased emergency water supply from the lower reaches of the Tarim River and its prolonged duration, the water area in the Taitema Lake area was continuously maintained at a larger state, expanding towards the northeast lowland.

Fig. 4 shows that from 2001 to 2020, as the Tarim River gradually extended to the southwest, the ecological risk along its banks gradually decreased and formed a low ecological risk corridor connecting with the Qarqan River. With the continuous implementation of EWC and increased water volume, the width of this corridor gradually expanded. The Tarim River and Taitema Lake water also provided a water source for the oases in front of the Altun Mountains. The barriers formed by the two low ecological risk corridors collectively prevented the expansion of the Taklimakan Desert and its merging with Lop Nur. At this time, the very high ecological risk areas in the study area decreased from 46.15 % in 2001 to 41.59 % in 2020, reaching 32.51 % and 34.59 % during the peak water supply years of 2005 and 2016, respectively. The very low ecological risk areas increased from 4.14 % in 2001 to 16.99 % in 2020.

### 3.3 Spatial clustering analysis of ERI

In 1986, 1991, 1995, 2001, 2005, 2009, 2016, and 2020, the global Moran's Index for the ERI was 0.761, 0.75, 0.737, 0.706, 0.709, 0.726, 0.681, and 0.688, respectively. The global Moran's Index remained positive for all eight years and exceeded 0.6. This indicates that there was a clear positive spatial autocorrelation in the ERI around the Taitema Lake area. Adjacent regions exhibited a high degree of spatial similarity. However, the overall spatial clustering of the ERI showed a declining trend. A local spatial autocorrelation analysis was conducted to understand the location of spatial clustering and the reasons behind the continuous decline in the global Moran's Index.

**Fig. 5:**
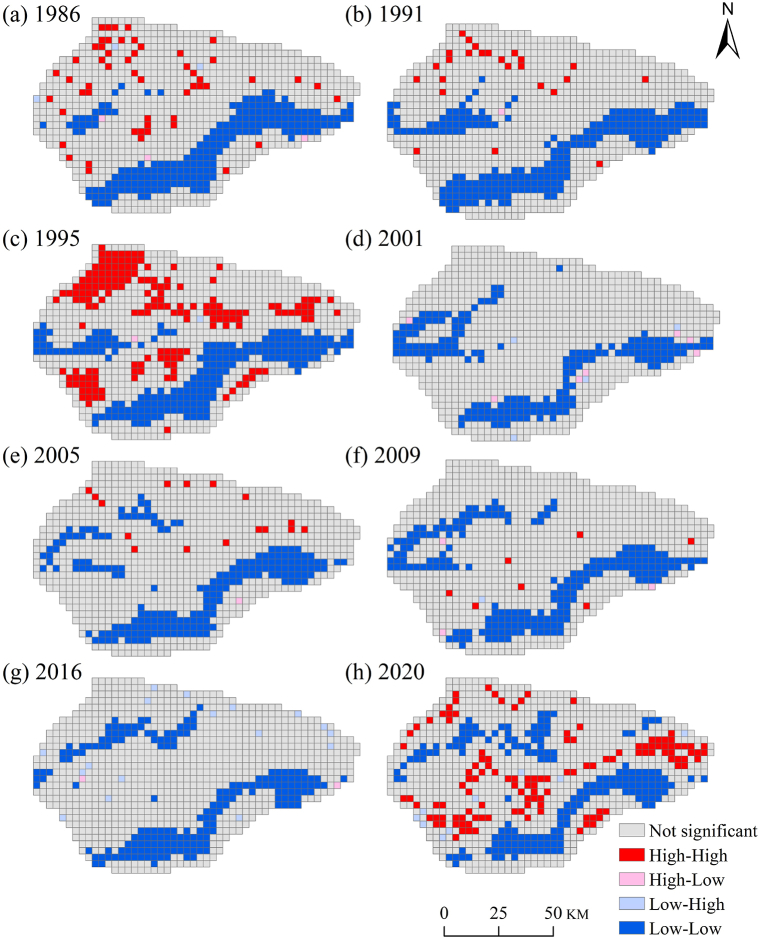
Local indicators of spatial association map of ecological risk in Taitema Lake region.

We observed that local spatial autocorrelation of the ERI in the study area was mainly characterized by H–H (high-high) and L-L (low-low) areas (see Fig. 5). Overall, L-L clustering areas were primarily distributed in the oases of the Altun Mountains' foothills and downstream of the Qanqan River. However, due to the dominance of areas with high ecological risk in the study area, there is not particularly strong spatial clustering in most years, with high-high clusters scattered throughout the study area. In 1995, due to the Qanqan River's course change and increased runoff, the areas affected by the Qanqan River gradually expanded, significantly reducing very high ERI areas within the study area. H–H clustering areas appeared in the northern part of the study area. With the conclusion of the Qanqan River's course change, in 2001, very high ERI areas reached their maximum during the study period (46.15 %). The H–H clustering areas gradually decreased and disappeared, and the regions with very high ERI once again became dominant. The L-L clustering areas downstream of the Qanqan River shifted northward, and the L-L clustering areas in the Altun Mountains' foothills reduced in size. Subsequently, under the influence of EWC from the Tarim River, the lower reaches of the Tarim River and Taitema Lake gradually recovered. The restored Taitema Lake wetlands, Kanglayka wetlands, and the Milan-Ruoqiang oasis broke the dominance of the very high ERI areas. H–H ERI clustering areas and L-L clustering areas exhibited a crossover pattern.

## 4 Discussion

### 4.1 Analysis of the impact of water bodies and towns on ERI

From the research results, we have observed that areas with low ERI are often found surrounding water bodies and towns, while areas far away from water bodies and towns typically have high ERI. To understand the impact of changes in water bodies and towns on ERI, we used Geographically Weighted Regression (GWR) to analyze the spatial pattern changes in the influence of water bodies and towns on ERI across the 34 years.

**Fig. 6:**
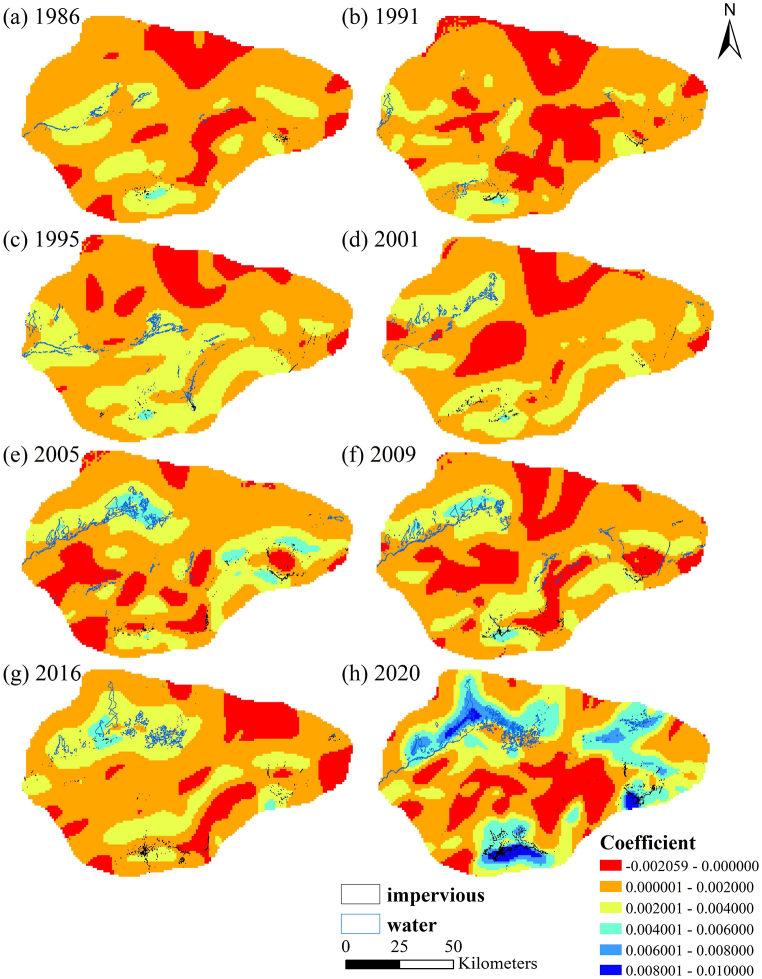
The impact coefficient of the distance to water/impermeable surfaces on the Landscape Ecological Risk Index.

In the early stages of the study, due to the increase in population and expansion of agricultural land in the upper reaches of Lake Taitema, as well as the annual decrease in downstream water flow, Lake Taitema, which serves as the tailwater lake of the Tarim River, has dried up. During this period, the study area only had water bodies in the downstream area of the Qarqan River and two seasonal rivers, the Ruoqiang River and the Milan River, with an annual runoff of less than 100 million cubic meters [40,41]. Urban areas play a role in water transmission within a certain distance. Within a distance of about 10 km from the water bodies and urban areas, there is a strong positive correlation between ERI and the distance from the water bodies and urban areas, indicating that the farther away from the water bodies and urban areas, the higher the ERI. After about 15 km, the impact significantly diminishes. However, in some downstream areas of rivers, due to unstable water levels, frequent drying up of water bodies may lead to increased salinity in water and surrounding soil. The higher salinity in water and soil has adverse effects on the growth of surrounding vegetation, leading to decreased landscape stability, fragmentation of patches, and consequently higher ERI in the surrounding areas. This is illustrated in Fig. 6 (a) by the red area downstream of the Milan River in the northwest of Milan town. Although there is residual water from the Milan River in the northern part, the ERI near the water surface area is higher. Subsequently, due to factors such as the diversion of the Qarqan River, fluctuations in water levels, the establishment of the Tarim River Ecological Water Conservancy Area (EWC), and the restoration of Lake Taitema, there were corresponding changes in the ERI in areas affected by rivers and urban areas.

We found that an increase in river and lake water volume and the duration of water bodies had a strengthening effect on the influence of water bodies on ERI in the surrounding areas. From Fig. 6 (h), it can be seen that by 2020, Taitema Lake had fully recovered and was connected to the Kanglayka Lakes, and even had connections to the Ruoqiang River and Milan River. At this point, the correlation coefficient between ERI and distance from water bodies increased from 0.002 to 0.004 in 1986 to 0.008–0.01. This demonstrates the change in the intensity of the impact of changes in water volume on ERI. From Fig. 6, it can also be seen that from 2001, when the Qarqan River's course change was completed, to 2020, the downstream of the Qarqan River and the Kanglayka Lakes had an increasing impact on the surrounding areas ERI, highlighting the importance of the duration of water bodies. The increase in the duration of water presence has two main effects. On one hand, it gradually replenishes groundwater, raising the water table and making it easier for vegetation in the area to access water. On the other hand, the original desert areas, with the presence of a stable water source, gradually transform into oases over a long-term process, with a consistent water supply, the quantity, diversity, and biomass of vegetation increase year by year. Both of these effects contribute to the gradual improvement of the oasis ecosystem, resulting in a decrease in ERI.

However, from Fig. 6, it can also be observed that the impact of large continuous water bodies on the surrounding ERI is often weaker than that of dispersed, fragmented water bodies. For example, in 1995, the impact of the lake formed by the water aggregation downstream of the Qarqan River was weaker than the dispersed water bodies in the middle and lower reaches of the Qarqan River and downstream of the Ruoqiang River. The large continuous water surfaces of the Kanglayka Lakes from 2005 to 2020, and the continuous water surfaces on the west side of the Taitema Lake, had a noticeably weaker impact on the surrounding ERI compared to the more dispersed water surfaces in the middle and lower reaches of the Qarqan River and the eastern side of the Taitema Lake, and even weaker than the impact of the Ruoqiang County on the surrounding ERI.

The factors contributing to this phenomenon can be mainly attributed to (1) Large continuous water bodies exhibit relatively monotonous landscape types. From the calculation formula of ERI, large water bodies occupy most of the area in a grid, resulting in a higher landscape dominance index. Additionally, water bodies rank second in landscape vulnerability among the five landscape types. The combination of high landscape dominance index and landscape vulnerability leads to a higher ERI. In arid regions and considering the characteristics of water bodies, the landscape diversity of large single landscape areas is low, and their resistance to external influences is also low. Moreover, water bodies at the edges of desert regions themselves are highly fragile, and their formation and disappearance depend entirely on the local degree of aridity and human water diversion. (2) Although large lakes have a considerable amount of water, their impact on vegetation growth and the surrounding ecological environment is limited. While lake water can replenish groundwater through seepage, it also has a certain impact radius. Previous studies have shown that a large amount of water supply does not necessarily lead to a high groundwater recharge rate. The correlation between runoff loss per kilometer and groundwater depth is stronger than the correlation with water supply[42]. In contrast, under the same amount of water, dispersed and fragmented water bodies have longer shorelines, resulting in a larger impact range and a greater impact on ERI. (3) Several lakes in the northern part of Kanglayka Lakes are surrounded by sand dunes, making the surrounding areas unsuitable for vegetation growth even if there is water. The water in these lakes tends to naturally dissipate or seep into the desert, resulting in no significant change in the surrounding ERI. (4) The study area has an annual evaporation rate exceeding 2000 mm. Additionally, Lake Taitema has a dish-like shape, with shallow water and an average depth of less than 1 m. However, the lake surface area is large, resulting in a very high evaporation rate. A large amount of water evaporates before being utilized by vegetation or infiltrating into the soil[42].

### 4.2 The benefits and prospects of EWC

Since the implementation of the EWC project in 2000, its effectiveness has been verified in multiple basins in the arid regions of northwest China. In addition to the Tarim River basin studied here, the Heihe River basin in Inner Mongolia, like the Tarim River, is located in an extremely arid area. Over the past 20 years, after the intensive agriculture in the middle reaches resulted in excessive exploitation of water resources, the downstream of the river gradually dried up, vegetation decreased, and ecological degradation occurred. Over the 15 years since the implementation of the EWC project, the water level in the downstream of the Heihe River has gradually risen, vegetation in the basin has recovered, and the NDVI value has increased from 0.10 to 0.13[11], with the terminal lake, Lake Juyan, reaching an area of over 50 km^2^[5]. In the Shiyang River located in the Hexi Corridor of Gansu Province, the downstream Qingtu Lake had completely dried up in the 1950s, forming a wind and sand stretching 13 km. The Badain Jaran Desert and the Tengger Desert encircle Qingtu Lake. The EWC project of the Shiyang River began in 2008, and by 2018, the lake area had recovered to 5.7 km^2^, and the ecological environment of the basin had been restored[43], effectively restraining the expansion trends of the two deserts[44]. Through scientifically unified management of basin water systems and timely and rapid replenishment of water to rivers and lakes in the middle and lower reaches, the EWC project plays an important role in improving the ecological environment in the middle and lower reaches of arid region rivers.

While improving the natural ecological environment of the region, the implementation of the EWC project has also provided favorable conditions for the development of regional agriculture and tourism. Before the implementation of the EWC project, due to the worsening natural conditions year by year, the upstream inflow of water decreased continuously, groundwater levels dropped continuously, and the salinity of drinking water for the downstream population of the Tarim River increased significantly. Moreover, the fluoride content exceeded standards, sandstorms were frequent, farmland was continuously buried by sand, and living conditions deteriorated continuously, leading to severe constraints on regional economic development, with per capita income below $170/a. The local government once proposed to relocate all residents here and resettle them elsewhere. After the implementation of the EWC project, the cultivated area of crops in the Tarim River basin has increased significantly since 2000, reaching a total increase of 73 % by 2020. According to statistics, in 1999 before the implementation of the EWC project, the GDP of Yuli County was only $32.8 million, with per capita income of $336 and an average cotton yield of 63 kg per mu. However, in 2007 after the implementation of the EWC project, the GDP increased to $300 million, with per capita income reaching $789 and an average cotton yield of 138 kg per mu. In the five towns downstream of the Tarim River, per capita income increased from $471 in 1998 to $1294 in 2007. The average cotton yield reached 181.5 kg, ranking first in the country for four consecutive years, and the actual sown area increased by 28 %. The number of livestock raised locally increased by 193 % compared to before the implementation of the EWC project, and the average stocking rate of grassland increased from 0.08 sheep/hm^2^ in 1999 to 1.23 sheep/hm^2^ in 2006[45,46].

**Fig. 7:**
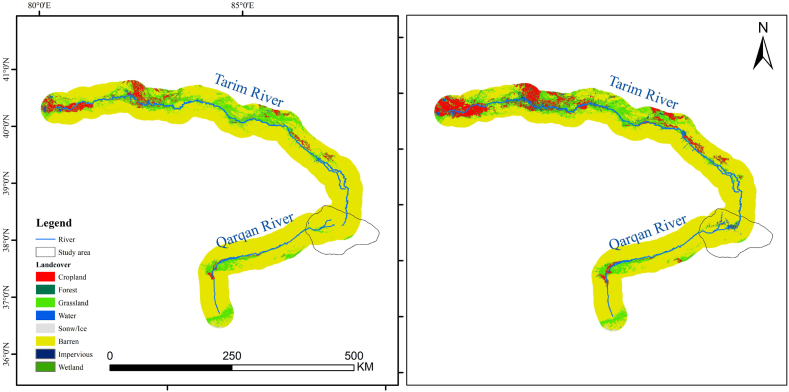
The landcover of Tarim River basin in 1986 and 2020.

**Fig. 8:**
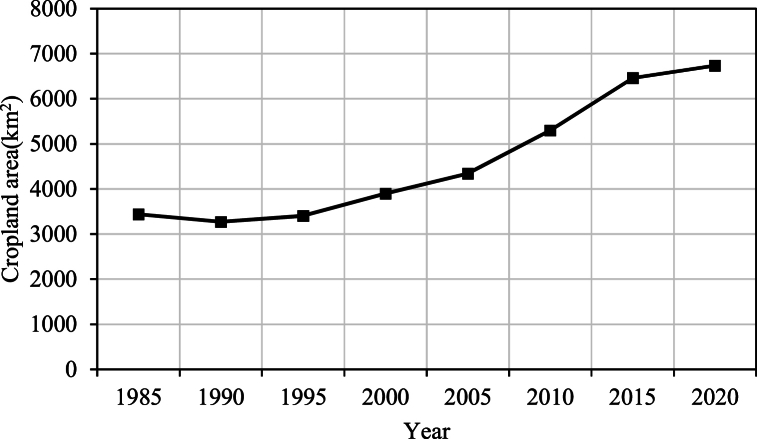
Changes in cultivated land area in the upper and middle reaches of the Tarim River and Qarqan River from 1985 to 2020.

During the 20 years of the EWC project implementation, the increase in the flow of the Tarim River not only significantly expanded the natural vegetation area, especially in the middle and lower reaches, but also augmented the available water for agricultural and pastoral use in the basin. This resulted in a substantial increase in cultivated land area, orchard area, and grazing volume within the basin. While the prosperity of agriculture, forestry, and animal husbandry brought economic income to the surrounding residents, it also led to a sharp rise in irrigation water demand. As depicted in Fig. 7, from 1985 to 2000, there were only minor fluctuations in the cultivated land area near the Tarim River and Qarqan River. However, starting from 2000, the area increased rapidly, reaching 6735.27 square kilometers by 2020, a 73 % increase compared to 2000. Additionally, as shown in Fig. 8, most of the increase in cultivated land was concentrated in the upper and middle reaches of the Tarim River. These areas were not water-deficient or experiencing only slight water shortages before the implementation of the EWC project. It was precisely because excessive water diversion in the upper reaches of the Tarim River exacerbated the shrinkage and drying up of the lower reaches that the EWC project was implemented. This has led to an “efficiency paradox"[47] in water resource utilization in the Tarim River basin. The implementation of ecological water transfer has stimulated more agricultural and pastoral water use, which not only contradicts the original intention of ecological water transfer but also exacerbates the contradictions in water resource utilization in the middle and lower reaches. The water volume of the EWC project mainly comes from the abundant tributaries in the upstream area of the Tarim River and the nearby Bosten Lake. Without controlling the expansion of agriculture and animal husbandry in the Tarim River basin and achieving a balance between water use and river-lake water volume, this project will not be able to sustain in the long term.

### 4.3 Ecological risk prevention measures based on landscape patterns

**Fig. 9:**
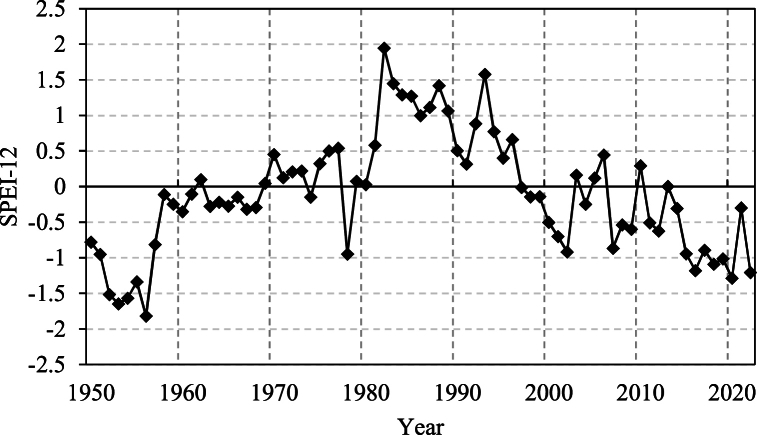
Variations in Standardized precipitation evaporation index (SPEI)-12 within the study area.

The Taitema Lake Basin between the Taklamakan Desert and the Lop Nur, along with the Ruoqiang-Milan Oasis in its south, collectively form an ecological barrier, preventing the expansion, spread, and merging of these two deserts. Being situated in one of the most arid regions of the Eurasian continent, with an annual average precipitation of 25 mm and an evaporation of 2500 mm. Fig. 9, depicting the standardized precipitation-evapotranspiration index (SPEI-12) at a 12-month scale for the study area, shows that for most years between 1950 and 2023, the annual mean SPEI-12 was less than 0. This indicates that the region has experienced a long-term arid climate with insufficient precipitation, with only a relatively humid period observed between 1980 and 1995. Therefore, in the absence of the EWC project implementation and without a significant increase in the runoff of the Qarqan River, the overall ERI of the study area still decreased from 1986 to 1995. During this period, the area with high and extremely high ERI decreased from 63.3 % in 1986 to 55.53 % in 1995. Additionally, the study area has been in a prolonged drought state, where vegetation growth, changes in landscape patterns, and ERI variations are entirely influenced by water bodies. The decline in ERI is mainly attributed to changes in the water volume of the Qarqan River and the influence of the Tarim River's EWC project water diversion.

1 The Qarqan River's course change from 1989 to 2009 led to the gradual northward movement of the low ERI areas in the Qarqan River's downstream landscape ecological risk[39,48]. This caused the gradual shrinkage of the Milan Oasis to the west of the study area and an increase in ERI.
2. After 2000, the increase in runoff in the Qarqan River led to a gradual decrease in ERI in the Qarqan River's downstream area, an expansion of areas with moderate to low ERI, the recovery and expansion of the adjacent Ruoqiang Oasis along the Qarqan River, and a reduction in ERI.
3. Starting in 2000, the EWC from the Tarim River provided ample water resources to the western part of the study area, resulting in the recovery of Taitema Lake and the expansion of the Milan Oasis. During this period, ERI significantly decreased, and an additional ecological barrier was formed, consisting of the Kanglayka Lakes and Taitema Lake, on the foundation of the existing Ruoqiang-Milan Oasis ecological barrier. This barrier not only increased resistance to the expansion of the Taklamakan Desert, building upon the foundation of the Ruoqiang-Milan ecological barrier but also provided an ecological barrier for the people of Ruoqiang County and Milan Town. This barrier reduced the harm from sandstorms originating in the Taklamakan Desert and the threats posed by shifting dunes to farmlands and urban areas.

However, we have also observed that the vegetation and ERI in the Taitema Lake basin and its surroundings are highly sensitive to changes in water bodies. Variations in river runoff and lake surface area can lead to significant fluctuations in ERI within a few months. Over the two decades of EWC, the increase in runoff in the Tarim River resulted in the substantial expansion of cultivated land and orchards in the upper and middle reaches of the Tarim River, leading to a sharp rise in irrigation water demand. Fig. 8 shows that the cultivated land area in the vicinity of the Tarim River and Qarqan River experienced only slight fluctuations from 1985 to 2000. However, starting from 2000, it increased rapidly and reached 6735.27 km^2^ by 2020, representing a 73 % increase compared to the year 2000. Additionally, the region's severe climate aridity and the uncertainty of water sources for EWC have added to the uncertainty of landscape ecological risk in the Taitema Lake basin.

Overall, the Tatom Lake basin still faces several challenges after experiencing drying and degradation followed by the EWC project's water diversion. Firstly, due to the diversion of the Cherchen River, its terminal lake, Konrak Lake, now extends deep into the Taklamakan Desert, where a significant amount of water is ultimately lost. In the future, it may be considered to construct hydraulic facilities and modify the river channel, allowing the downstream flow of the Cherchen River to shift slightly southward, generating greater ecological benefits and alleviating the pressure on the Tarim River's ecological water sources. This would help strengthen and consolidate the ecological barrier between the Cherchen River and the Tarim River. Additionally, measures such as desert soil stabilization could be implemented to alter the desert topography around Konrak Lake. Planting vegetation suitable for desert conditions can reduce the area's ERI while also conserving water and soil through reduced water dissipation and enhanced infiltration.

Secondly, although the ecological water brought in by the EWC project has facilitated the restoration of natural vegetation around the river channels, it has also promoted the expansion of farmland and orchards in the middle and upper reaches of the Tarim River. The contradiction between ecological and economic water use has not been alleviated and even shows a trend of exacerbation. In future urban planning, the planting area of farmland and orchards in the Tarim River basin should be restricted. There is a need to change the inherent belief among grassroots departments and the public that “planting vegetation in the desert can reduce or even eliminate desertification.” The Tarim River basin has long been in a state of extreme drought, with almost all water sources coming from glacier meltwater. Once the regional water balance is disrupted, all oases on the eastern edge of the Taklamakan Desert will be threatened. Policymakers must not consider the ecosystem and human society separately but systematically consider the interaction between humans and nature, balance economic and ecological water use, in order to achieve sustainable development of the regional ecology.

Thirdly, the existing water delivery modes of the EWC project mainly include river channel water delivery and natural overflow. Although this mode effectively raises the groundwater level in the vicinity and has a significant effect on the restoration of surrounding natural vegetation growth, its impact range is very limited. Multiple research results have found that the influence of river channels on surrounding vegetation does not exceed 1 km and the impact on surrounding ERI does not exceed 10 km, with a severe attenuation as the distance increases. The water in the natural overflow area is very shallow but covers a large surface area, resulting in significant evaporation and dissipation of water. In the future, controlling the inflow into the lake and adding shading covers to river channels and reservoirs can reduce water loss. At the end of the river, ecological gates, and natural overflow areas, combining river network dense with water delivery modes such as sprinkler irrigation, drip irrigation, and pipelines can expand the impact area of ecological water under the same water volume, maximizing the ecological benefits of water.

### 4.4 Limitations of this study

This study focuses on investigating the impact of ecological water conveyance (EWC) on landscape ecological risks in terminal lake areas of arid regions. However, due to the lack of on-site monitoring data, remote sensing data were utilized to extract water area and land cover data in the study area. In the future, improving data accuracy could be achieved by on-site measurements, and additional depth data for water bodies could be incorporated. Measurement and long-term monitoring of groundwater levels could also aid in elucidating the specific impact mechanisms of EWC projects on surrounding vegetation. These aspects can be further enriched and expanded upon in future research.

## 5 Conclusion

This study evaluated the ERI in the Taitema Lake Basin from 1986 to 2020, analyzed the changes in ERI and its driving factors, investigated the influence of the Ecological Water Conveyance (EWC) project on ERI, and provided suggestions for the further implementation of the EWC project from the perspective of landscape ecological risk. The results indicate:

1. Over the past 20 years of the implementation of EWC project, the water area of Taitema Lake has gradually increased and connected with the Kanglayka Lake group, resulting in a total area increase from 72.5 km^2^ to 199.6 km^2^. The desert area within the region has decreased by 731 km^2^, while the grassland area has increased by 509 km^2^. The overallERI in the study area has significantly decreased, leading to the reestablishment of the oasis ecological barrier downstream of the Tarim River, thereby preventing the expansion of the Taklimakan Desert and Lop Nur.
2. The ERI in the study area exhibits strong spatial clustering, with a significant decrease observed in the ERI around water bodies and urban areas, albeit with limited influence range. By altering the water conveyance methods and modifying downstream river channels, it will be possible to improve the regional landscape pattern and expand the range of landscape ecological low-risk areas.
3. The contradiction between regional socio-economic water usage and ecological water usage still persists. We recommend that policymakers fully consider the interaction mechanisms between human activities and the ecological environment during the policy formulation stage. By balancing the contradiction between ecological and economic water usage, long-term sustainable development of watershed management can be achieved.

## Funding

This study was supported by the Third Xinjiang Scientific Expedition and Research Program—Investigation and Risk Assessment of Aeolian Disasters in Tarim Basin (No. 2021xjkk0305).

## CRediT authorship contribution statement

**Zhentao Lv:** Writing – review & editing, Writing – original draft. **Shengyu Li:** Visualization, Supervision. **Xinwen Xu:** Supervision. **Jiaqiang Lei:** Supervision. **Zhongmin Peng:** Data curation.

## Declaration of competing interest

The authors declare that they have no known competing financial interests or personal relationships that could have appeared to influence the work reported in this paper.
